# Meeting Global Health Needs via Infectious Disease Forecasting: Development of a Reliable Data-Driven Framework

**DOI:** 10.2196/59971

**Published:** 2025-03-21

**Authors:** Ravikiran Keshavamurthy, Karl T Pazdernik, Colby Ham, Samuel Dixon, Samantha Erwin, Lauren E Charles

**Affiliations:** 1 Artificial Intelligence and Data Analytics Division, Pacific Northwest National Laboratory, 902 Battelle Boulevard, Richland, WA, 99354, United States; 2 Paul G. Allen School for Global Health, Washington State University, Pullman, WA, United States; 3 Department of Statistics, North Carolina State University, Raleigh, NC, United States

**Keywords:** disease forecasting, machine learning, deep learning, epidemiology, One Health, decision-making, data visualization

## Abstract

**Background:**

Infectious diseases (IDs) have a significant detrimental impact on global health. Timely and accurate ID forecasting can result in more informed implementation of control measures and prevention policies.

**Objective:**

To meet the operational decision-making needs of real-world circumstances, we aimed to build a standardized, reliable, and trustworthy ID forecasting pipeline and visualization dashboard that is generalizable across a wide range of modeling techniques, IDs, and global locations.

**Methods:**

We forecasted 6 diverse, zoonotic diseases (brucellosis, campylobacteriosis, Middle East respiratory syndrome, Q fever, tick-borne encephalitis, and tularemia) across 4 continents and 8 countries. We included a wide range of statistical, machine learning, and deep learning models (n=9) and trained them on a multitude of features (average n=2326) within the One Health landscape, including demography, landscape, climate, and socioeconomic factors. The pipeline and dashboard were created in consideration of crucial operational metrics—prediction accuracy, computational efficiency, spatiotemporal generalizability, uncertainty quantification, and interpretability—which are essential to strategic data-driven decisions.

**Results:**

While no single best model was suitable for all disease, region, and country combinations, our ensemble technique selects the best-performing model for each given scenario to achieve the closest prediction. For new or emerging diseases in a region, the ensemble model can predict how the disease may behave in the new region using a pretrained model from a similar region with a history of that disease. The data visualization dashboard provides a clean interface of important analytical metrics, such as ID temporal patterns, forecasts, prediction uncertainties, and model feature importance across all geographic locations and disease combinations.

**Conclusions:**

As the need for real-time, operational ID forecasting capabilities increases, this standardized and automated platform for data collection, analysis, and reporting is a major step forward in enabling evidence-based public health decisions and policies for the prevention and mitigation of future ID outbreaks.

## Introduction

The frequency and magnitude of infectious disease (ID) events have seen a drastic incline in the past few decades, mainly attributed to climate change, urbanization, and globalization [1]. These events often involve the emergence of novel infectious agents or the re-emergence of a previously known pathogen. Timely and accurate prediction of such ID events is crucial for decision makers to decrease associated mortality, morbidity, and economic losses [2]. However, the complex and unpredictable nature of pathogen ecology makes forecasting the spatiotemporal dynamics of IDs a challenging task [3].

Digital information related to ID events is being generated and shared faster than ever before. Additionally, information associated with disease occurrence, such as meteorology, socioeconomics, demographics, land use, agriculture, social media, and internet trends, is often readily available [4]. To keep up with this unprecedented amount of data being generated, machine learning (ML)– and deep learning (DL)–based algorithms are being adopted by the research community. These methods have shown to be better at detecting cryptic patterns arising from interactions between multiple features, which are difficult, often impossible at times, to uncover with conventional prediction methods [5].

The time-series models have been previously used for forecasting ID events including brucellosis [6], Middle East respiratory syndrome [7], campylobacteriosis [8], and Q fever [5]. However, over the last decade, especially after the COVID-19 pandemic outbreak, considerable progress has been made in the field of ID surveillance, as the disease diagnosis, reporting, and intelligence-sharing infrastructure continue to grow on a global scale [9]. Dixion et al [5] compared the performance of various forecasting approaches across several diseases and countries and found that tree-based techniques had better predictive performance compared to statistical and DL techniques. Integrating diverse large-scale epidemiological data from multiple sources has further enhanced the accuracy and utility of disease prediction models [10]. Ensemble forecasting techniques, which merge predictions from multiple models, offer notable advantages over single-model approaches [11]. For example, Reich et al [12] used real-time multimodel ensembles for seasonal influenza in the United States, while Ma et al [13] demonstrated the effectiveness of integrating internet search data along with ensemble forecasting techniques to jointly forecast COVID-19 and influenza-like illnesses, underscoring the value of diverse data sources in public health forecasting.

Despite several advantages, ML and DL techniques do come with a number of limitations, making them less desirable in the real-world operational setting. We conducted a scoping systematic review to determine the status and advances made in the field of ID prediction using ML and DL, which were published elsewhere [9]. One of our focuses in this systematic review was to identify if and how researchers were incorporating the necessary methods required to eventually enable the operational deployment of their ID forecasting models. Through this work, we identified multiple shortcomings in published modeling techniques that could hinder their ability to support operational biopreparedness and decision-making. Even though most of the studies focused on increasing the prediction accuracy of their models, which is critical for operational deployment, they did not address other important decision-making metrics such as computational efficiency, uncertainty quantification, interpretability, and generalizability. Modern ML and DL techniques are performance-driven, that is, they aim to generate better predictive or classification accuracy by minimizing errors [14]. As a result, other metrics that are crucial for operational decision-making are often overlooked by the scientific community. First, as the structural and functional complexities of the forecasting models evolve, the computational resources required to train such models also grow considerably. In addition, these complexities result in an expanded number of model hyperparameters that can be individually tuned using numerous possible techniques. This ever-increasing complexity gives rise to an even larger problem space with highly variable results. Second, since most of these techniques are nonparametric in nature, model uncertainties are not inherently estimated. Hence, model uncertainties are often overlooked even though these estimates are crucial for systematic and transparent decision-making [15]. Third, ML and DL models are considered black boxes, meaning that their internal logic and inner workings are mostly hidden from the end user [9]. Consequently, verifying and understanding the rationale behind the model forecasts are difficult and often neglected. With the above considerations in mind, we developed an ID forecasting pipeline that holistically focuses on these often neglected yet essential performance metrics required for operational decision support. By incorporating this pipeline into an interactive dashboard, we created a capability that allows users to easily visualize the ID forecasting results for use in operational planning and decision-making.

## Methods

### Overview

**Figure 1.**
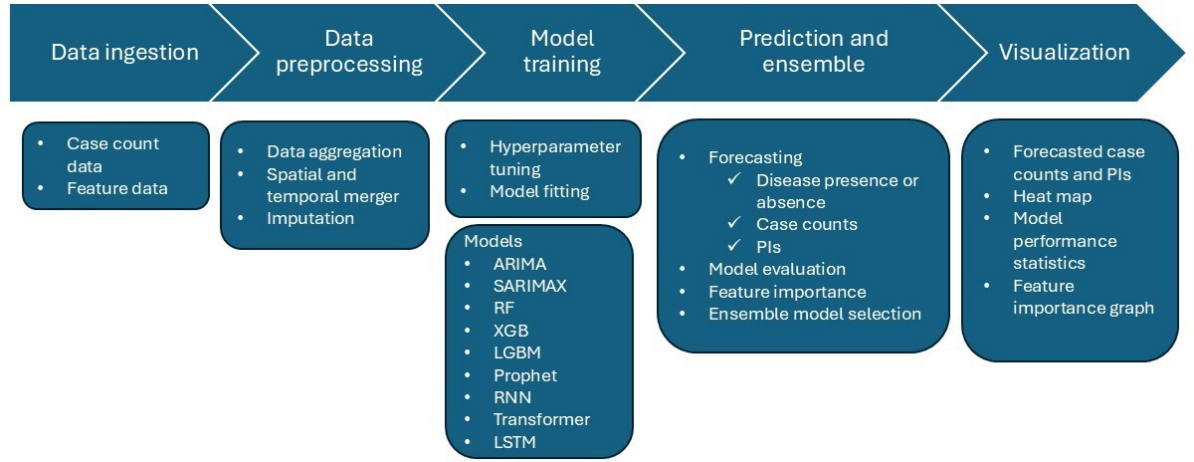
Graphical flowchart illustrating the infectious disease forecasting pipeline. ARIMA: autoregressive integrated moving average; LGBM: light gradient boosting machine; LSTM: long short-term memory; PI: prediction interval; RF: random forest; RNN: recurrent neural network; SARIMAX: seasonal autoregressive integrated moving average with exogenous factors; XGB: extreme gradient boosting.

The graphical flowchart illustrating our forecasting pipeline including data ingest, preprocessing, model training, prediction, and visualization is presented in Figure 1.

### Case Count Data

The model outcome variable was case counts collected from EpiArchive [16] and Food and Agriculture Organization’s Emergency Prevention System-I [17]. We included the most consistent, available, and interpretable disease case counts from these sources. These data also included locations that reported only 0 disease cases. In instances where only the disease presence was reported without any information about the exact number of cases, the region, or the date of occurrence, such disease-location combinations were excluded from the analysis. We included all the diseases that spanned across multiple countries or regions and had a consistent reporting of disease events for our study period. Data were collected at the regional-level resolution for each country from January 2014 to December 2018, which contained case counts at either the weekly or monthly temporal resolution.

### Feature Data

We collected feature data from a variety of open sources across the One Health landscape (Table 1). Broadly, the features included historic case counts (case count lags up to 6 months), country census data, socioeconomic factors, health data, agricultural trade data, landscape, and climate records. The feature information varied in spatial and temporal resolutions; data were typically available at monthly or yearly resolutions and covered either the country or regional level. A complete list of countries, regions, and the total number of features collected for each region is presented in Multimedia Appendix 1.

**Table 1.** The input feature data types, source, feature description by name, geographic location, geographic resolution, time period, and periodicity. The table was originally published by Dixon et al [5].

Data type	Individual features	Geographic location	Geographic resolution	Time period	Periodicity
Case counts [16]	Incidences of select human diseases	Countries of interest	Region level	2009-2018	Daily
Political borders [18]	Geopolitical borders (country and within the country)	Countries of interest	Region level	2018	Single instance
Climate [19,20]	Air temperature, humidity, precipitation, soil moisture, and wind speed	Global	Gridded 0.25 °×0.25 °, 1 °×1 °	2012‐2018	Monthly
Gross domestic product [21]	Gross domestic product	Global	Country level	Varies	Yearly
Elevation [22]	Digital elevation map	Global	43,200×17,200 (30 arc seconds)	N/A^a^	N/A
Mortality [23]	Deaths by country, year, sex, age group, and cause of death	Global	Country level	2009‐2018	Yearly
Municipal waste [24]	Municipal waste generation and treatment	Countries of interest	Country level	2009‐2017	Yearly
Sociopolitical and physical data [25]	Socioeconomic and political attributes	Global	Varies by country; 1: 10-110 m	2019	Single instance
Population [26]	Population by age intervals by location	Global	Country level	2009‐2015	Every 5 years
Population density [27]	Population density	Global	30 arc seconds	2009‐2015	Every 5 years
Water potability and treatment [24]	Freshwater resources, available water, wastewater treatment plant capacity, surface water	Countries of interest	Country level	2009‐2017	Yearly

a N/A: not applicable.

### Data Preparation and Imputation

The harvested raw data contained information at varied spatial and temporal resolutions in different file formats. These raw data were harmonized to create the final datasets containing monthly case counts and input features for each region within the country. In situations where the feature data at the regional level were missing, the features aggregated by an average at the national level were used. Any features with more than 20% missing values across all regions and dates were not included as a predictor variable. The remaining missing values were imputed temporally using spline and forward-fill techniques for monthly and yearly feature data, respectively. Some features could not easily be imputed temporally through these techniques but were still sufficiently prevalent across regions to include in the models. In these instances, the majority of the missing data (less than 20% overall) came from only a few select regions. For these special cases, we performed geoimputation using the k-nearest neighbor method and restricted input to only regional feature data within the same country. We used distance-based weights for the features with 5 neighboring samples for imputation. The imputation techniques were implemented using the “pandas” and “skforecast” libraries in Python (Python Software Foundation) [26].

### Model Choice

#### Overview

In our analysis, we included the following models discussed in detail below: statistical time-series models (ie, autoregressive integrated moving average [ARIMA], seasonal autoregressive integrated moving average with exogenous factors [SARIMAX], and Prophet), tree-based models (ie, random forest [RF], extreme gradient boosting [XGB], and light gradient boosting machine [LBGM]), and DL models (recurrent neural network [RNN], long short-term memory [LSTM], and transformer).

#### Statistical Time-Series Models

The ARIMA model is a traditional statistical technique used in time-series forecasting. These models use a linear, regression-type equation, in which the predictors are lags of the dependent variable or lags of the forecast errors. The SARIMAX is an extension of ARIMA that takes seasonal trends into account within time-series data. Because SARIMAX can also accommodate exogenous features, it is often preferable to traditional ARIMA. However, SARIMAX models may fail to converge if those exogenous features are highly correlated. To overcome this issue, the least absolute shrinkage and selection operator (LASSO) regression was used for feature selection and dimensionality reduction. These LASSO-selected features were later used as input variables for SARIMAX. The modeling techniques were implemented using “statsmodels” and “sklearn” libraries in Python [28,29].

Prophet is a decomposable time-series model that uses 3 main components to make its forecasts, namely, trend, seasonality, and holidays [30]. The trend component models the nonperiodic changes in the time series. Seasonality models the periodic, or seasonal, changes in the time series; this can be an important part of a disease forecasting model, as many diseases have seasonal trends. The Prophet model was implemented using the “darts” library in Python [31].

#### Tree-Based ML Models

Tree-based models make predictions by creating decision trees that divide the feature space using a series of binary decision thresholds. The RF is an extension of the decision tree method that uses an ensemble of decision trees to increase performance and reduce the risk of overfitting the training data. The XGB is an implementation of a decision tree that uses stochastic gradient boosting to sequentially improve prediction during training. Because of this methodology, XGB achieves superior accuracy in prediction tasks. LGBM is similar to XGB; however, it also applies a novel sampling method and feature bundling process that allows the method to get comparable performance to XGB but uses far less memory and trains much faster. These modeling techniques were implemented using “skforecast” library in Python [32].

#### DL Models

The RNN is built on DL architecture with a hidden state that memorizes sequential data. This added process provides the model with an understanding of temporal information in a time-series sequence. The LSTM is an extension of RNN with additional parameters that incorporate different scales of temporal information, which allow the model to effectively use both long- and short-term temporal data in its prediction. Hence, these models are routinely used in disease forecasting tasks.

Transformers [33] are another form of DL architecture that has been recently outperforming other models in many applications of ML. These models leverage a structure known as “attention,” which allows them to model dependence in both short and long temporal lags. Unfortunately, these models are especially slow to train and often require very large amounts of data and computational power. The DL techniques were implemented as an encoder-record architecture using “darts” library in Python.

### Model Training and Prediction

The model training involved hyperparameter tuning and model fitting, whereas model testing involved generating 1-step ahead forecasts (ie, monthly) and their prediction intervals (PIs). When creating predictions, all feature data and disease case counts up until the time of prediction were included in the model. The model training included hyperparameter tuning using a 5-fold time-series cross-validation split and model fitting. First, the models were trained using 2014‐2016 data and tested on 2017 data. Subsequently, the model was retrained using 2014‐2017 data and tested on 2018 data in a sliding window manner. A detailed description of model-specific hyperparameters and their search space used for model training is presented in Multimedia Appendix 1.

### Model Evaluation

The forecasting models were evaluated in two ways: (1) *F*_1_-score to evaluate the ability of a model to predict the presence or absence of a disease in each region (ie, did the region report 1 or more cases during the testing period) and (2) mean absolute error (MAE) for the subset of predictions where the disease was detected in a region (case counts greater than 0). The multimetric approach was adopted because of the high frequency of time-location pairs that had no disease present. Without separating these results, the MAE metric was largely driven by the results of the high-frequency disease and regions. Finally, we created ensemble models by selecting the best-performing technique based on the MAE of the testing dataset. Other metrics such as precision, recall, negative predictive value, true negatives, false positives, false negatives, and true positives were also estimated but not considered when building our ensemble model.

### Uncertainty Quantification and Interpretability

The 95% PIs for our forecasted values were estimated to account for forecasting uncertainties. For statistical models (ARIMA, SARIMAX, and Prophet), PIs were readily obtained along with the model forecasts as a natural consequence of the model construction. However, PIs were not directly available for ML and DL techniques. Hence, we used Python packages that retroactively produced PIs using alternate techniques. The PIs for tree-based models (RF, XGB, and LGBM) were estimated by a bootstrapping technique using “skforecast” library, whereas for DL models (RNN, LSTM, and transformer), PIs were calculated by a nonparametric method known as quantile regression using the “Darts” library [31,32]. For each model forecast, we estimated the coverage probability of the 95% PIs (ie, instances where the PIs surrounded the true value) as a measure of calibration assessment for the prediction uncertainties.

Feature importance, based on Shapley Additive Explanation (SHAP) values for the best-performing models, was used as a generalized approach to interpretability for tree-based and DL models [34]. SARIMAX was the only statistical model that allowed for feature input and interpretability, which was provided by coefficient estimates from the model. However, the SARIMAX model was unable to incorporate the large number of features available for each region. To address this limitation, a feature selection approach was applied using LASSO regression to reduce the model’s input variables.

The historic case count data are important and most frequently used predictive features in the ID prediction domain [9]. In our analysis, we used case count lags up to 6 months. To estimate the relative importance of these case count lags compared to other features, we calculated 2 metrics, namely, the feature ratio and mean reciprocal rank (MRR). A threshold of the top 10 features, as deemed by the models, was set to estimate the feature ratio and MRR. The feature ratio was the total number of lag case count features divided by the total number of features (up to the top 10 features). The MRR, on the other hand, also considers the position of the first relevant item in the ranked list, that is, case count lags. The MRR was defined as the mean of reciprocal ranks of case count lags across all features. The values range from 0 to 1, with a higher score signifying the greater relative feature importance rank of case count lags compared to other features for obtaining model forecasts.

### Spatial Generalizability Using Transfer Learning

We used the transfer learning framework to test the models for their ability to produce accurate forecasts in regions that were fairly new to the disease. These regions were strategically picked by maximizing the global coverage and following the stratified random sampling framework. First, only the country-disease combinations where case count data were available were selected. This process also included the regions that reported 0 disease cases during our study period. Next, one region within the selected countries was randomly picked and denoted as the target region. Then, we selected a similar region to the target region to train the transfer learning models based on (1) the target and similar region being part of the same country and (2) the target and similar region containing comparable case counts over the last 6 months. The similar region pretrained model was then used to forecast the disease case counts of the target region, and performance was assessed.

### Ethical Considerations

Data used in this study are open source and do not identify individual information, either directly or indirectly. Therefore, this research was exempted from ethical review.

## Results

### Overview

The summary statistics of the disease-location combinations aggregated at the country level are presented in Table 2, whereas a detailed breakdown of this summary for both test and train split is shown in Multimedia Appendix 1.

**Table 2.** Median (IQR) regional case counts per country and disease for the entire dataset from January 2014 to December 2018.

Disease	Country							
Australia, median (IQR)	Germany, median (IQR)	Israel, median (IQR)	Japan, median (IQR)	Norway, median (IQR)	Saudi Arabia, median (IQR)	Sweden, median (IQR)	United States, median (IQR)
Brucellosis	3 (0-29)	11 (0-42)	30 (0-1072)	0 (0-3)	0 (0-2)	—^a^	1 (0-2)	4 (0-25)
Campylobacteriosis	7904 (0-43,205)	148,616 ( 50,644-197,616)	998 (0-7666)	—	306 (0-1456)	—	1103 (0-10,815)	1159 (0-25,327)
MERS^b^	0 (0-0)	—	—	0 (0-0)	—	3 (0-71)	—	—
Q fever	55 (0-974)	28 (0-769)	46 (0-103)	0 (0-1)	—	—	—	5 (0-26)
Tick-borne encephalitis	—	15 (0-897)	—	0 (0-2)	—	—	—	—
Tularemia	—	—	0 (0-0)	0 (0-1)	15 (0-69)	—	—	4 (0-89)

a Not available.

b MERS: Middle East respiratory syndrome.

There were 757 disease-region combinations ran through 9 different models in this study. As the first step, we calculated *F*_1_-scores to determine the ability of the forecasting models to detect the presence or absence of a disease in a given region (Table 3). Overall, tree-based models (ie, XGB, RF, and LGBM) and Prophet consistently had better and comparable *F*_1_-scores across the diseases. The DL models (ie, RNN, transformer, and LSTM) had significantly lower *F*_1_-scores. Patterns were consistent across all diseases in each region. The additional evaluation metrics for model and disease combination including precision, recall, negative predictive value, true negatives, false positives, false negatives, and true positives are presented in Multimedia Appendix 1.

**Table 3.** *F*_1_-score for all models forecasting the presence or absence of disease for each region in 2017 and 2018.

Disease	Model								
	ARIMA^a^	LGBM^b^	LSTM^c^	Prophet	RF^d^	RNN^e^	SARIMAX^f^	Transformer	XGB^g^
Brucellosis	0.65	0.67	0.55	0.69	0.67	0.56	0.66	0.59	0.67
Campylobacteriosis	0.93	0.96	0.96	0.97	0.96	0.95	0.91	0.94	0.96
MERS^h^	0.98	0.90	0.60	0.90	0.90	0.61	0.95	0.56	0.90
Q fever	0.78	0.78	0.64	0.79	0.78	0.67	0.74	0.65	0.78
Tick-borne encephalitis	0.68	0.98	0.59	0.97	0.98	0.55	0.95	0.54	0.98
Tularemia	0.54	0.73	0.49	0.71	0.73	0.50	0.72	0.55	0.73

a ARIMA: autoregressive integrated moving average.

b LGBM: light gradient boosting machine.

c LSTM: long short-term memory.

d RF: random forest.

e RNN: recurrent neural network.

f SARIMAX: seasonal autoregressive integrated moving average with exogenous factors.

g XGB: extreme gradient boosting.

h MERS: Middle East respiratory syndrome.

Next, we assessed the performance of each model for the subset of regions, where the total case counts were greater than 0 (ie, the disease was present) based on MAE. The model with the lowest MAE was inconsistent across diseases (Table 4).

**Table 4.** MAE^a^ (95% CI) for each disease aggregated across all locations where the disease was present in 2017 and 2018.

Disease	Model								
	ARIMA^b^, MAE (95% CI)	LGBM^c^, MAE (95% CI)	LSTM^d^, MAE (95% CI)	Prophet, MAE (95% CI)	RF^e^, MAE (95% CI)	RNN^f^, MAE (95% CI)	SARIMAX^g^, MAE (95% CI)	Transformer, MAE (95% CI)	XGB^h^, MAE (95% CI)
Brucellosis	0.8 (0.5‐1.1)	0.8 (0.5‐1.1)	0.84 (0.5‐1.14)	0.9 (0.5‐1.2)	0.8 (0.5‐1.1)	0.9 (0.5‐1.2)	1.1 (0.7‐1.6)	0.8 (0.5‐1.6)	0.8 (0.4‐1.1)
Campylobacteriosis	237.9 (1.1‐474.9)	43.8 (35.3‐52.3)	45.2 (36.3‐54.12)	43.4 (34.9‐51.8)	39.8 (32.3‐47.2)	46.6 (37.7‐55.5)	243.7 (74.9‐412.6)	50.9 (40.3‐61.6)	43.1 (34.2‐52.1)
Q fever	28.9 (25.4‐83.1)	1.5 (0.9‐2.1)	1.18 (0.8‐1.6)	1.4 (0.9‐1.9)	1.5 (0.8‐2.1)	1.3 (0.9‐1.8)	1.9 (1.2‐2.7)	1.4 (0.9‐2.0)	1.2 (0.9‐1.5)
Tick-borne encephalitis	2.0 (0.6‐3.3)	2.4 (0.7‐4.1)	2.61 (0.7‐4.53)	1.3 (0.5‐2.2)	2.4 (0.6‐4.1)	2.6 (0.7‐4.6)	1.6 (0.6‐2.6)	2.9 (0.7‐5.2)	2.4 (0.6‐4.1)
Tularemia	0.6 (0.4‐0.7)	0.6 (0.5‐0.7)	0.58 (0.5‐0.71)	0.6 (0.5‐0.7)	0.6 (0.5‐0.7)	0.6 (0.4‐0.7)	0.7 (0.5‐0.9)	0.6 (0.5‐0.7)	0.6 (0.5‐0.7)

a MAE: mean absolute error.

b ARIMA: autoregressive integrated moving average.

c LGBM: light gradient boosting machine.

d LSTM: long short-term memory.

e RF: random forest.

f RNN: recurrent neural network.

g SARIMAX: seasonal autoregressive integrated moving average with exogenous factors.

h XGB: extreme gradient boosting.

The MAE of the best-performing model for each disease across all locations (with case counts greater than 0) is presented in Table 5, which we define as our “ensemble” model. Using this technique and when averaging across all regions, prediction years, and diseases, we observed that the ensemble model had the lowest MAE compared to other models (Table 6). While the difference does not appear to be a large amount, the difference is considerable, and the magnitude is visually minimized by the large error in the ARIMA and SARIMAX models. All the models included in our study had similar MAE except for ARIMA and SARIMAX since they had a few outlier predictions that skewed their MAE.

**Table 5.** Ensemble (best performing) models for each disease averaged across all regions where the disease was present in 2017 and 2018.

Disease	Best model	MAE^a^ (95% CI)
Brucellosis	LGBM^b^	0.8 (0.5‐1.1)
Campylobacteriosis	RF^c^	39.8 (32.3‐47.2)
Q fever	LSTM^d^	1.2 (0.8‐1.6)
Tick-borne encephalitis	Prophet	1.4 (0.5‐2.2)
Tularemia	ARIMA^e^	0.6 (0.4‐0.7)

a MAE: mean absolute error.

b LGBM: light gradient boosting machine.

c RF: random forest.

d LSTM: long short-term memory.

e ARIMA: autoregressive integrated moving average.

**Table 6.** MAE^a^ (95% CIs) for each model aggregated across all diseases and regions where the disease was present in 2017 and 2018.

Model	MAE (95% CI)
ARIMA^b^	115.2 (5.2‐225.2)
Ensemble	8.7 (6.5‐23.9)
LGBM^c^	20.8 (16.5‐25.1)
LSTM^d^	21.4 (16.9‐25.9)
Prophet	20.6 (16.3‐24.9)
RF^e^	19.0 (15.2‐22.8)
RNN^f^	22.1 (17.6‐26.7)
SARIMAX^g^	113.1 (34.6‐191.6)
Transformer	24.2 (18.8‐29.5)
XGB^h^	20.5 (16.0‐25.0)

a MAE: mean absolute error.

b ARIMA: autoregressive integrated moving average.

c LGBM: light gradient boosting machine.

d LSTM: long short-term memory.

e RF: random forest.

f RNN: recurrent neural network.

g SARIMAX: seasonal autoregressive integrated moving average with exogenous factors.

h XGB: extreme gradient boosting.

**Figure 2.**
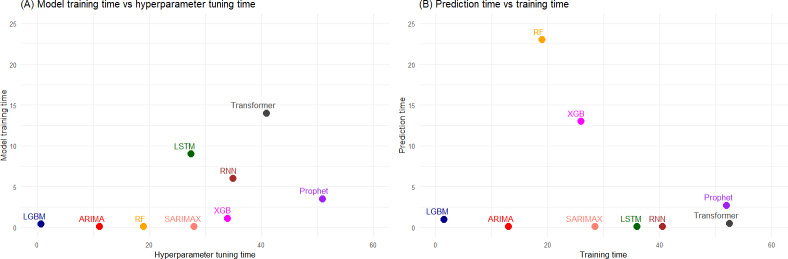
(A) Average hyperparameter tuning and model fitting time and (B) average training and prediction time for each model averaged across all diseases, locations, and periods. ARIMA: autoregressive integrated moving average; LGBM: light gradient boosting machine; LSTM: long short-term memory; RF: random forest; RNN: recurrent neural network; SARIMAX: seasonal autoregressive integrated moving average with exogenous factors; XGB: extreme gradient boosting.

The average training time, which included hyperparameter tuning and model fitting, along with prediction time (ie, the time to generate 1-step ahead forecasts and their PIs) for each model averaged across all diseases, regions, and periods are presented in Figure 2. The LGBM followed by ARIMA were the most time-efficient models. Although RF and XGB had a training time comparable with other models, their average prediction time was considerably higher mainly because of the increased amount of time required by them to generate 95% PIs using the bootstrapping technique.

### Feature Importance

**Figure 3.**
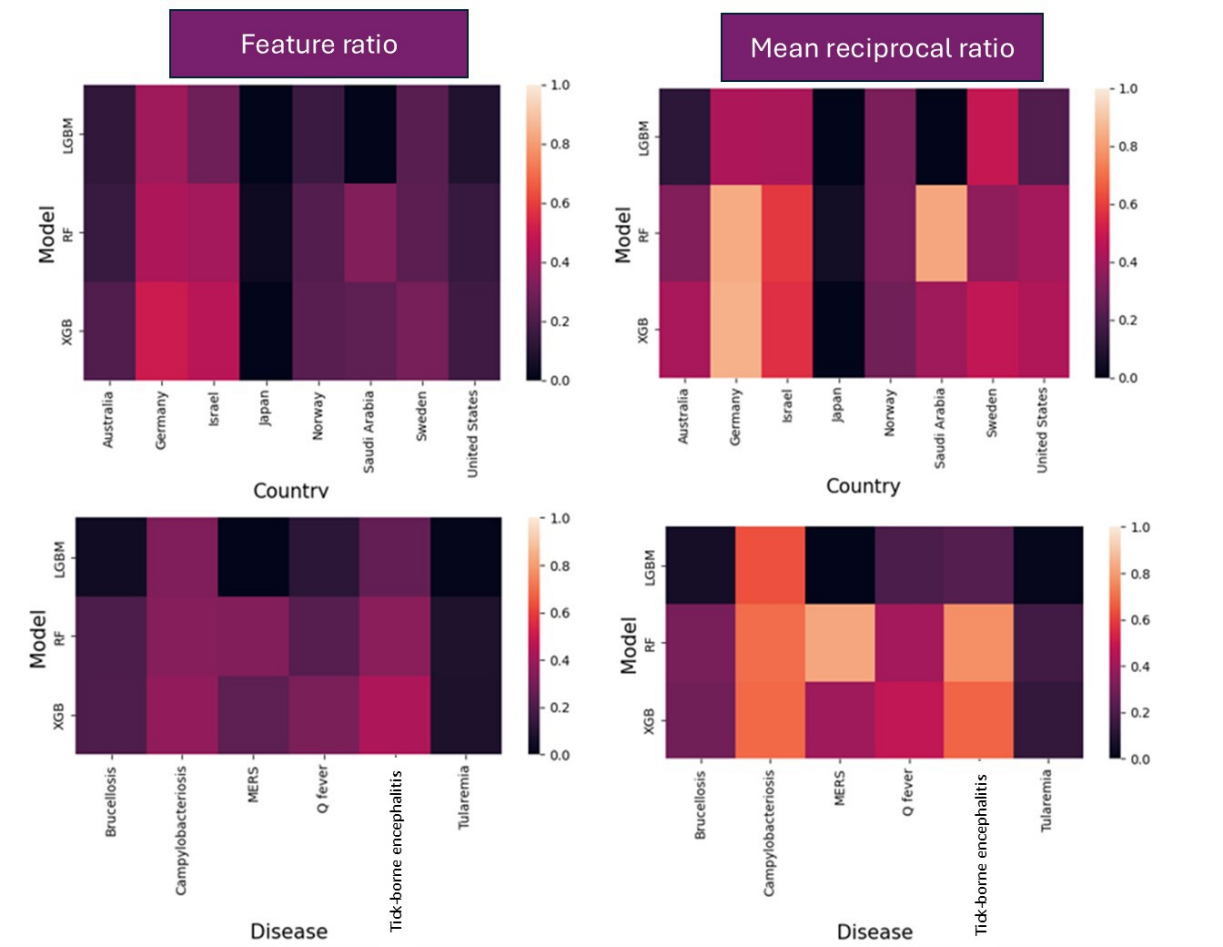
Relative importance of case count lags to produce forecasts for tree-based models quantified as feature ratios and mean reciprocal ranks of the most important features (up to the top 10) aggregated across countries (top row) and diseases (bottom row). Higher scores signify the greater relative importance of case count lags compared to other features for obtaining model forecasts. LGBM: light gradient boosting machine; MERS: Middle East respiratory syndrome; RF: random forest; XGB: extreme gradient boosting.

For the tree-based models (ie, LGBM, RF, and XGB), we selected up to 10 top features based on SHAP values that the model deemed important and calculated feature ratio and MRR to show how lag case count may be impacting our predictive performance (Figure 3). Overall, the feature ratios were low (<0.4) when aggregated across countries and diseases, suggesting that non–lag case count features were more important for forecasting compared to case lag data. Campylobacteriosis and tick-borne encephalitis had higher MRR compared to other diseases, indicating that lag case count features were ranked relatively higher among the top 10 features for these diseases. However, the low feature ratio and MRR for many diseases highlight how the additional feature variables can be valuable in disease forecasting, especially for less prevalent diseases. A complete breakdown of feature ratios and MRRs for each country-disease combination is presented in Multimedia Appendix 1.

The coverage probability of the forecasting models represented by the percent coverage of the true observations by 95% PIs is presented in Table 7. The LGBM had a 94.4% (n=14,707) coverage, indicating that the PIs were well calibrated, even better than traditional statistical models like ARIMA and SARIMAX. On the other hand, the DL models had less than 10% coverage, highlighting the fact that these models were not thoroughly calibrated. This observation was true when the coverage percentage was broken down across disease and country combinations in Multimedia Appendix 1.

**Table 7.** Coverage percentage of the 95% prediction intervals of the forecasting models^a^.

Model	Coverage, n (%)
ARIMA^b^	14,582 (93.6)
LGBM^c^	14,707 (94.4)
LSTM^d^	1277 (8.2)
Prophet	13,086 (84)
RF^e^	13,756 (88.3)
RNN^f^	1246 (8)
SARIMAX^g^	13,959 (89.6)
Transformer	1262 (8.1)
XGB^h^	13,491 (86.6)

a The ideal coverage percentage is 95%.

b ARIMA: autoregressive integrated moving average.

c LGBM: light gradient boosting machine.

d LSTM: long short-term memory.

e RF: random forest.

f RNN: recurrent neural network.

g SARIMAX: seasonal autoregressive integrated moving average with exogenous factors.

h XGB: extreme gradient boosting.

### Results Visualization

**Figure 4.**
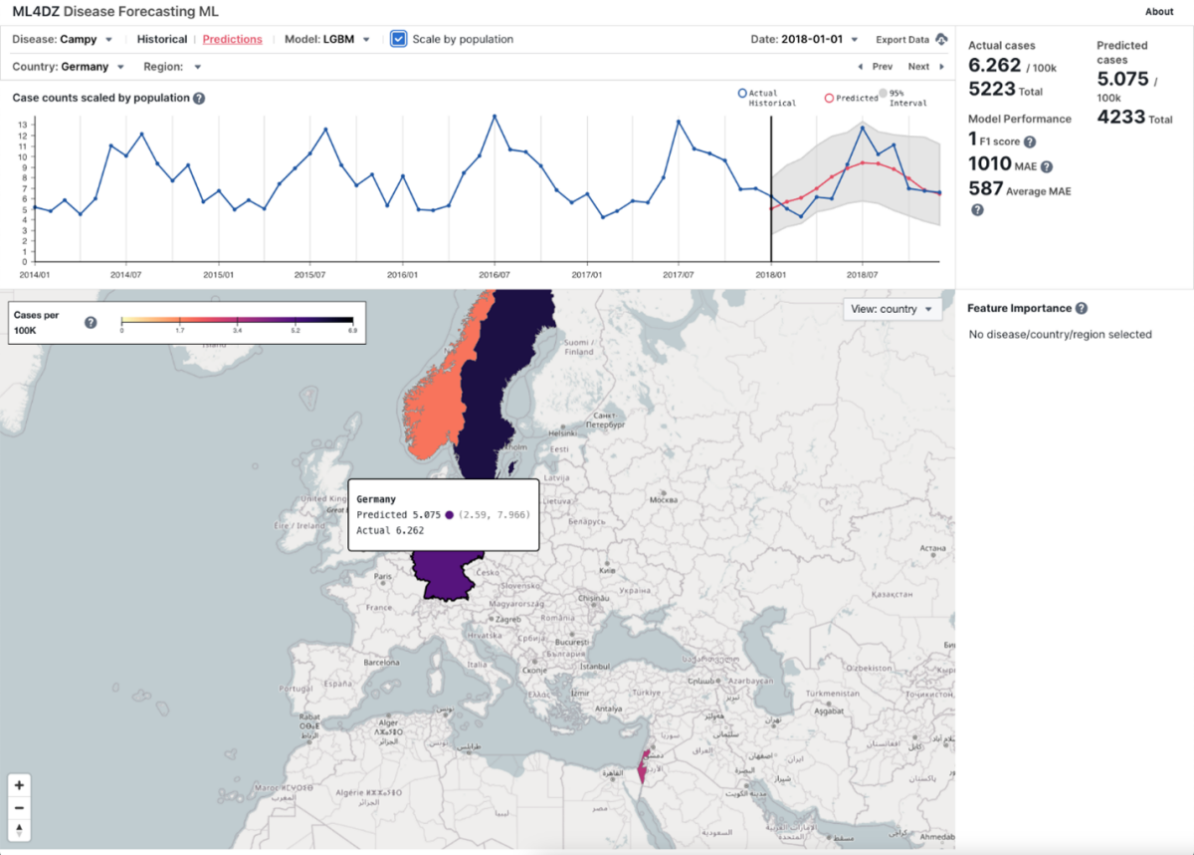
Dashboard in prediction view with values based on case counts scaled by population. LGBM: light gradient boosting machine; MAE: mean absolute error; ML: machine learning.

**Figure 5.**
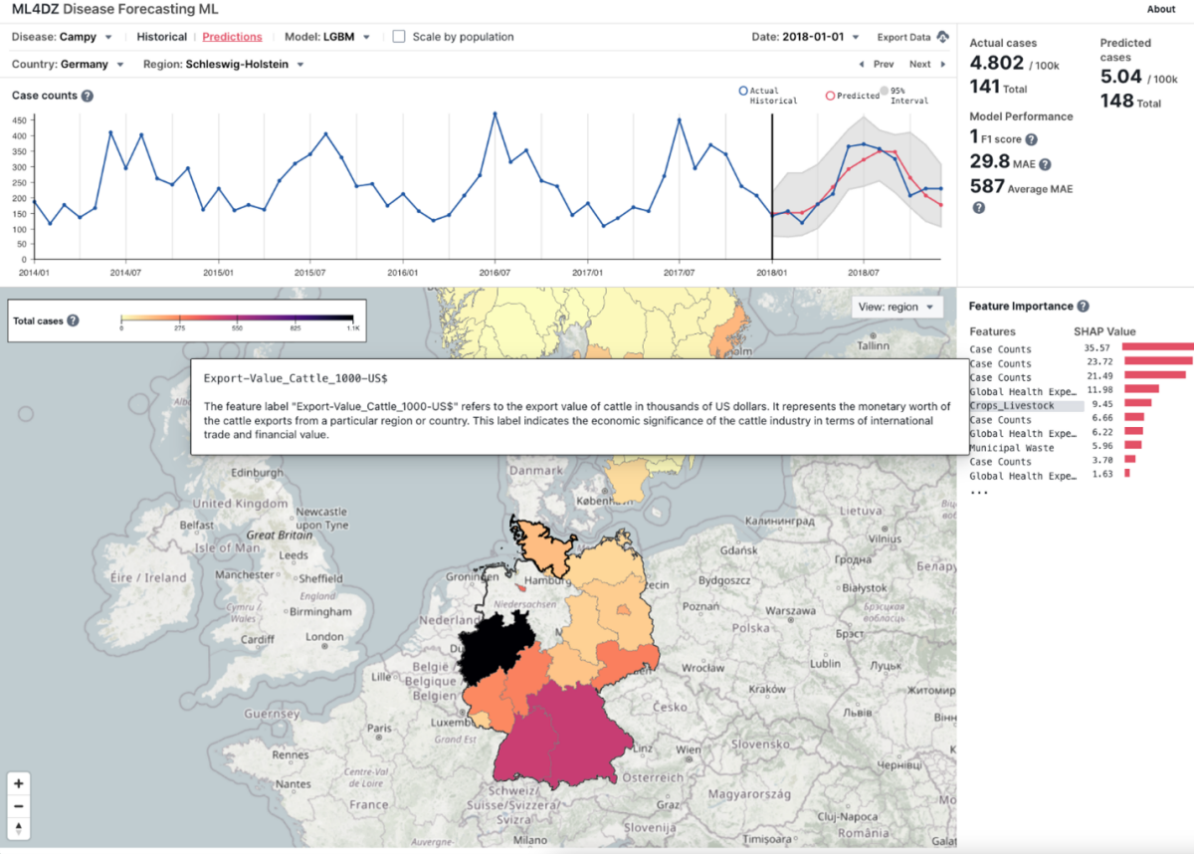
Dashboard with disease (“campy”=campylobacteriosis) and region (Schleswig-Holstein, Germany) selected in prediction view, which includes model performance statistics in summary and model feature importance list. Pop-out box provides a detailed explanation of the clicked and highlighted feature. LGBM: light gradient boosting machine; MAE: mean absolute error; ML: machine learning.

We created an interactable dashboard to visualize the predictions made by forecasting models (Figure 4). The information presented in the user interface includes a time series of actual and predicted values with 95% PI, model performance metrics, and feature importance values for each disease and country or region. In Figure 5, campylobacteriosis in Schleswig-Holstein, Germany, for the year 2018 using the LGBM model is presented as an example, showing uncertainty quantification by PIs and feature importance as mean SHAP values. The lagging case count feature for the past 6 periods was the most important feature for the model, but variables related to global health spending and monetary export value of cattle also ranked high in feature importance.

### Spatial Generalizability Using Transfer Learning

The spatial generalizability for the forecasting models was tested based on the performance of the pretrained ensemble model from the location with a comparable disease pattern (ie, similar region) to forecast the disease case counts of the selected region (ie, target region). The model tested (ensemble or best model) varied by location and included statistical (ARIMA: n=3) and tree-based models (LGBM: n=2 and XGB: n=1). In most cases, the MAE of a similar region was close to and often lower than the MAE of the target region (Table 8). However, campylobacteriosis forecasting in Kalmar, Sweden, was an exception, where the model MAE increased from 5.01 to 16.98 when Västmanland feature data were used for training. It is important to note that these are pairwise comparisons of a single metric calculated across a single region and do not include confidence bounds to determine if any differences are statistically significant. However, given that over half of the disease-region models produced an equal or lower MAE when testing in a new region, if a power analysis was conducted assuming a binary outcome and a continued trend, no amount of data would be sufficient to show that the transfer learning results would be significantly worse.

**Table 8.** The mean absolute error (MAE) values representing spatial generalizability of transfer learning models^a^.

Disease	Country	Target region(cases in the past 6 months)	Ensemble model	Similar region(cases in the past 6 months)	MAE ofbaseline forecast	MAE oftransfer learning forecast
Brucellosis	Israel	Jerusalem (34)	LGBM^b^	Akko (32)	5.41	2.3
Campylobacteriosis	Sweden	Kalmar (122)	XGB^c^	Västmanland (124)	5.01	16.98
MERS^d^	Saudi Arabia	Bisha (0)	ARIMA^e^	Eastern Province (0)	0.03	0.02
Q fever	Australia	New South Wales (37)	ARIMA	Queensland (48)	2.85	2.00
Tick-borne encephalitis	Germany	Saxony-Anhalt(3)	ARIMA	Brandenburg (3)	0.20	0.25
Tularemia	Japan	Ibaraki (0)	LGBM	Saga (0)	0.00	0.00

a For baseline forecasts, the models were trained and tested on target regions. For transfer learning forecasts, the models were trained on similar regions and tested on target regions.

b LGBM: light gradient boosting machine.

c XGB: extreme gradient boosting.

d MERS: Middle East respiratory syndrome.

e ARIMA: autoregressive integrated moving average.

## Discussion

### Principal Findings

As ML and DL techniques gain popularity in the ID forecasting domain, they are being extensively used across a wide range of pathogens with diverse ecology, geographic, and temporal scales. However, there is limited consensus among the forecasting community regarding the application and reporting of these results in a manner that lends themselves useful for operational decision-making in real-world circumstances [9]. In this study, we present a universal pipeline for analyzing data, generalizing the results, and automating reporting. We also address crucial operational metrics such as forecasting accuracy, computational efficiency, spatiotemporal generalizability, uncertainty quantification, and interpretability, which are essential in an operational scenario. This generalized and automated analytic pipeline is a major step toward addressing operational demands in the ID forecasting domain and to better inform public health and veterinary policies. We included 6 IDs with diverse transmission dynamics spanning 8 countries and 213 regions. Additionally, we trained our models using a broad range of data from demographic, geographic, climatic, and socioeconomic factors within the One Health landscape. This comprehensive approach enables our analysis to capture the intricate interplay of variables that drive infectious disease presence and transmission, more closely reflecting the complex realities observed in the real world.

### Model Performance

We assessed forecasting model accuracies by their ability to detect the presence of a disease based on *F*_1_-scores and by their ability to accurately forecast case counts when present using MAE. This 2-step approach prevented the inflation of model accuracy by the regions that did not encounter disease presence during our study period. Though we did not find a single best model suitable for all disease, region, and country combinations, the tree-based models were consistently better than all other models at detecting both disease presence and actual case counts with better *F*_1_-scores and MAE values, respectively. Our ensemble technique, which selects the best-performing model for each disease-location pair, demonstrates superior forecasting performance by reducing prediction noise compared to single-model approaches, aligning with previous research [35-37]. While ideal ensemble methods would dynamically update to identify the best model at each time stamp, this demands extensive data, computational resources, and continuous validation. To address these challenges, we opted for a simpler approach by selecting the best model based on MAE for the testing dataset, minimizing resource and data requirements. However, ensemble techniques still require substantial computational power and analytic pipelines, posing challenges for low- and middle-income countries with limited resources. Investments in infrastructure, skill development, and data-sharing initiatives are crucial to fully leverage ML and DL techniques for ID forecasting in such settings.

### Computational Efficiency

The computation time required for ID forecasting can broadly be split into model training and model prediction time. In our analysis, much of the training time was spent on hyperparameter tuning. Autoregressive and tree-based models took less time to tune than DL models. On the other hand, tree-based models (ie, RF and XGB) took considerably longer to produce predictions compared to the other models. This increase in time was mainly because of the time required to compute bootstrap PIs. The LGBM was an exception, as it used the least computational time for both training and testing compared to all the other models. Since our forecasting pipeline performed stepwise forecasting for each month with models retrained only once a year, more time was spent on predicting rather than model training. Hence, when building a forecasting pipeline where the models are retrained once a year and predictions are made every month, it is computationally optimal to choose models with lower prediction time rather than training time.

The accuracy of modeling techniques can drastically differ with the amount of time and computational resources spent on fine-tuning the model. Here, we chose the initial hyperparameter values for optimization based on their relative importance and time required for overall analysis with the goal of achieving comparable results. It is important to find a balance between model complexity and computational efficiency according to the individual operational needs and available resources.

### Uncertainty Quantification

Since the effectiveness of health policies and operational decision-making is driven by the accuracy of forecasts, policy makers need to know the credibility of the models in the form of prediction uncertainties [38]. In traditional statistical techniques, such as ARIMA and SARIMAX, forecasting uncertainties are computed based on probability theory and a set of statistical assumptions and, therefore, are readily available. However, generating such PIs is rather complicated in ML and DL compared to traditional methods due to additional uncertainties associated with noise distributions, hyperparameters, overparameterization, and optimization that should be accounted for [39]. Therefore, most of the ID forecasting studies fail to report their model uncertainty despite their recent popularity [9]. In our study, we used bootstrapping and probabilistic methods to compute PIs around our point estimates of ML and DL methods. Such bounds are crucial to assessing future uncertainties, making operational decisions, planning policies for a range of possible outcomes, and comparing different forecast models thoroughly [40]. The LGBM was the best-calibrated model with almost 95% of true observations covered by 95% of PIs, followed by other tree-based and statistical models. The DL models had the least desirable coverage along with their inferior predictive performance and narrow prediction band. This may not be surprising, though as there is evidence in the literature that uncertainty estimates around DL predictions often fail to capture the true data distribution [41].

### Spatiotemporal Generalizability

Overall, the predictive performance of transfer learning models was comparable to their base models as presented in Table 8. For example, using the Akko model to predict case counts of brucellosis in Jerusalem created an increase in predictive accuracy. This suggests the potential for spatial generalizability of our ensemble, that is, an ability to predict existing diseases in new regions with low error. However, campylobacteriosis forecasting in Kalmar, Sweden, was an exception, where the model MAE increased when Västmanland feature data were used for training. Since campylobacteriosis tends to have higher case counts, the difference in MAE cannot be directly comparable to the other diseases with much lower case counts and naturally lower MAE values. There are many possible reasons for the difference seen in the results. For example, the similar region model may not have contained the necessary similarities to the target region required to enable a lower prediction error, such as differences in regional management practices. The ensemble model (best-performing model for disease-location pair) was different between the test cases and included both statistical (only case counts) and tree-based (many features) models. More advanced transfer learning techniques that include multiple input features to identify similarity and emphasize model retraining by reallocating model weights between target and similar regions could be considered to improve spatial generalizability [2,42]. Regardless, these results are encouraging, and a more comprehensive study is warranted.

### Model Interpretability

We included interpretability for each model in the form of feature importance, a critical metric that is often neglected in the ID forecasting domain [9]. Interpretability provides insight into the decision-making process of the prediction systems, which is crucial for implementing appropriate disease mitigation strategies. Our scoping systematic review of ID prediction models showed that historic case counts are the most commonly used input feature in the ID forecasting domain, while the other important predictors, such as climate, demographics, socioeconomics, and geography, are often neglected [9]. In this study, we estimated lower feature ratio and MRR for lag case count features across countries and diseases, indicating that the non–case count features were equally important, if not more, compared to case count lags. This study suggests that including data related to the full disease ecology is critical for obtaining accurate, reliable, and interpretable ID forecasts. For example, cattle export was one of the important informative non–case count features for forecasting campylobacteriosis in Schleswig-Holstein, Germany. A direct causal association between the disease and cattle exports cannot be made just by a feature importance plot. However, campylobacteriosis is the most commonly reported bacterial food-borne gastrointestinal infection in the European Union that is closely associated with the dairy industry [43]. Our results suggest that high dairy and other agriculture activities, including cattle trading in Schleswig-Holstein, could play an important role in the disease prevalence in the region and, if adjusted, could result in disease mitigation.

### Data Visualization

To make the best-performing models and corresponding predictions accessible to decision makers, we developed an interactive data visualization dashboard. This dashboard provides the raw data, the model predictions with accompanying 95% PIs and feature importance, overall model performance, and an interactive global map. This dashboard allows a user to visualize forecasts for up to a year into the future in any country or region where the data exist and can be used to inform control measures to reduce the spread of an ID within a country or region.

### Conclusions

Our study provides a generalized platform to analyze and report ID forecasts with an emphasis on analytical accuracy, computational efficiency, uncertainty quantification, interpretability, and generalizability. These 5 aspects are crucial in determining the forecasting approach optimal for each situation’s operational needs. While all the forecasting techniques come with their own strengths and weaknesses, choosing an optimal approach is usually a tradeoff between computational efficiency, model complexity, and forecasting accuracy. Recognizing and addressing such nuances will facilitate the use of ID forecasts in an operational environment for better preparedness and response during an ID emergency.

## Supplementary material

10.2196/59971 Multimedia Appendix 1 Supplementary figures and tables.

## Abbreviations

ARIMA: autoregressive integrated moving average
DL: deep learning
ID: infectious disease
LASSO: least absolute shrinkage and selection operator
LGBM: light gradient boosting machine
LSTM: long short-term memory
MAE: mean absolute error
ML: machine learning
MRR: mean reciprocal rank
PI: prediction interval
RF: random forest
RNN: recurrent neural network
SARIMAX: seasonal autoregressive integrated moving average with exogenous factors
SHAP: Shapley Additive Explanation
XGB: extreme gradient boosting
