# Supplementary material for: Meeting Global Health Needs via Infectious Disease Forecasting: Development of a Reliable Data-Driven Framework
Source: JMIR Public Health Surveill. 2025 Mar 21;11:e59971. doi: 10.2196/59971 (PMC11951818; doi:10.2196/59971)
Supplement: Multimedia Appendix 1 [file publichealth-v11-e59971-s001.docx]

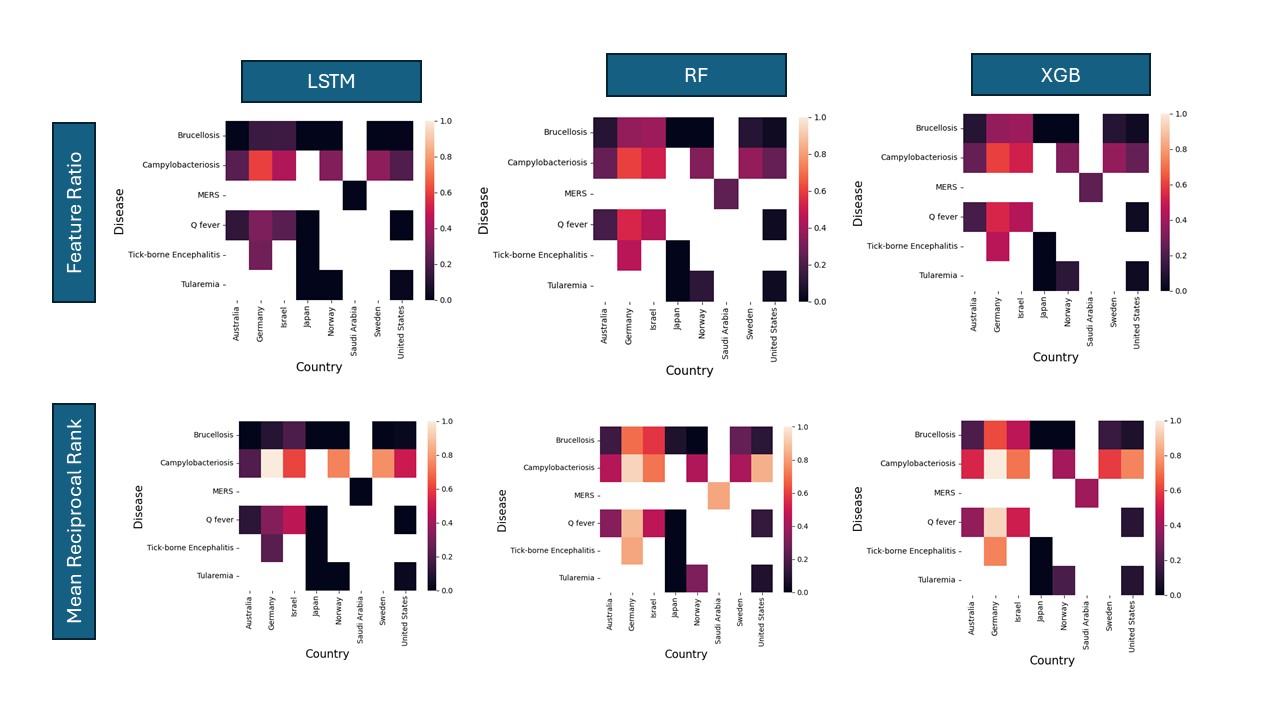


**Figure S1.** Feature ratio and Mean Reciprocal Rank for each country-disease combination.


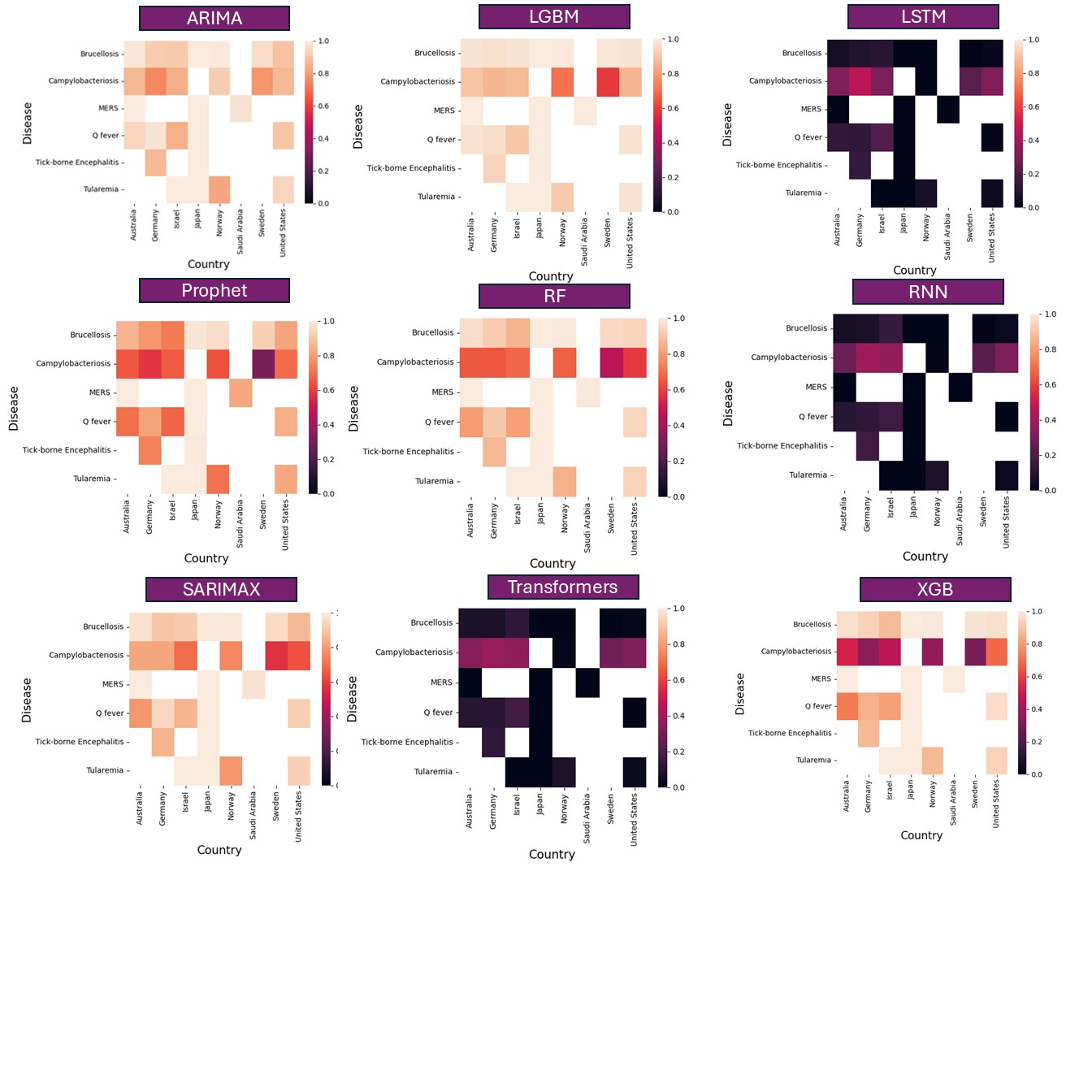


**Figure S2**. Coverage percentage of the 95% PIs of the forecasting models across countries and diseases. The lighter color indicates higher PI coverage. The countries where the disease information was absent are left blank (white).

**Table S1.** Description of model-specific hyperparameters and their search space.

| **Model** | **Python library** | **Hyperparameter** | **Search Space** |
| --- | --- | --- | --- |
| **ARIMA** | statsmodels | p | [0, 1, 2, 3, 4, 5, 6, 7] |
|  |  | q | [0, 1, 2, 3, 4, 5, 6, 7] |
|  |  | d | [0,1, 2] |
| **LGBM** | skforecast | n_estimators | [100, 250, 500] |
|  |  | max_depth | [10, 20, 30] |
|  |  | learning_rate | [0.1, 0.01] |
|  |  | num_leaves | [20, 30] |
| **LSTM** | darts | input_chunk_length | [6,12] |
|  |  | learning_rate | [1e-4, 1e-3, 1e-2] |
|  |  | output_chunk_length | [1] |
| **Prophet** | darts | growth | ['flat', 'linear'] |
|  |  | n_changepoints | [10, 20, 50, 75] |
|  |  | seasonality_mode | ['multiplicative','additive'] |
| **RF** | skforecast | n_estimators | [50, 100, 250] |
|  |  | min_samples_leaf | 1, 2, 4] |
|  |  | max_features | ['auto', 'sqrt'] |
|  |  | min_samples_split | [5, 10] |
| **RNN** | darts | input_chunk_length | [6,12] |
|  |  | n_rnn_layers | [1, 2] |
|  |  | batch_size | [24, 36] |
|  |  | hidden_dim | [50, 100] |
|  |  | learning_rate | [1e-4, 1e-3, 1e-2] |
| **SARIMA** | statsmodels | p | [0, 1, 2, 3, 4, 5, 6, 7] |
|  |  | q | [0, 1, 2, 3, 4, 5, 6, 7] |
|  |  | d | [0,1, 2] |
|  |  | P | [0,1] |
|  |  | Q | [0,1] |
|  |  | D | [0,1] |
|  |  | S | 12 |
| **Transformer** | darts | output_chunk_length | [1, 6, 12] |
|  |  | input_chunk_length | [6, 12] |
|  |  | learning_rate | [1e-4, 1e-3, 1e-2] |
| **XGB** | skforecast | max_depth | [2, 3, 6] |
|  |  | min_child_weight | [2,3, 6] |
|  |  | n_estimators | [100, 250] |
|  |  | learning_rate | [0.01, 0.1] |
|  |  | subsample | [0.7] |
|  |  | colsample_bytree | [0.7] |
|  |  | gamma | [0, 2, 4] |

**Table S2.** Median (Max) regional case counts per country and disease for the model training set from January 2014 to December 2016 and testing set from January 2017 to December 2018 (train/test).

| **Disease** | **Country** | | | | | | | |
| --- | --- | --- | --- | --- | --- | --- | --- | --- |
|  | **Australia** | **Germany** | **Israel** | **Japan** | **Norway** | **Saudi Arabia** | **Sweden** | **United States** |
| **Brucellosis** | 3 (20) / 0 (33) | 6 (42) / 4 (20) | 21 (740) / 11 (362) | 0 (6) / 0 (1) | 0 (2) / 0 (3) | - | 0 (14) / 0 (4) | 2 (35) / 2 (14) |
| **Campylo-bacteriosis** | 4659 (21879) /  3245 (14827) | 9199 (60346) / 5663 (37626) | 642 (4441) / 305 (2687) | - | 296 (1078) / 11 (72) | - | 798 (6876) / 307 (2836) | 570 (11453) / 434 (14136) |
| **MERS** | 0 (0) / 0 (0) | - | - | 0 (0) / 0 (0) | - | 3 (68) / 0 (0) | - | -- |
| **Q-fever** | 37 (680) / 9 (239) | 16 (660) / 10 (81) | 25 (64) / 29 (86) | 0 (1) / 0 (2) | - | - | - | 3 (16) / 1 (8) |
| **TBE** | - | 8 (425) / 7 (457) | - | 0 (1) / 0 (3) | - | - | - | - |
| **Tularemia** | - | - | 0 (0) / 0 (0) | 0 (1) / 0 (0) | 4 (16) / 11 (42) | - | - | 3 (44) / 1 (41) |

**Table S3.** List of countries, regions, and the total number of features collected for each region included in the study.

| Country | Region | Feature count |
| --- | --- | --- |
| Australia | Australian Capital Territory | 2332 |
| Australia | New South Wales | 2332 |
| Australia | Northern Territory | 2332 |
| Australia | Queensland | 2332 |
| Australia | South Australia | 2332 |
| Australia | Tasmania | 2332 |
| Australia | Victoria | 2332 |
| Australia | Western Australia | 2332 |
| Germany | Baden-Württemberg | 2546 |
| Germany | Bavaria | 2546 |
| Germany | Berlin | 2546 |
| Germany | Brandenburg | 2546 |
| Germany | Bremen | 2546 |
| Germany | Hamburg | 2546 |
| Germany | Hesse | 2546 |
| Germany | Lower Saxony | 2546 |
| Germany | Mecklenburg-Vorpommern | 2546 |
| Germany | North Rhine-Westphalia | 2546 |
| Germany | Rhineland-Palatinate | 2546 |
| Germany | Saarland | 2546 |
| Germany | Saxony | 2546 |
| Germany | Saxony-Anhalt | 2546 |
| Germany | Schleswig-Holstein | 2546 |
| Germany | Thuringia | 2546 |
| Israel | Afula | 2084 |
| Israel | Akko | 2084 |
| Israel | Ashqelon | 2084 |
| Israel | Beer Sheva | 2084 |
| Israel | HaSharon | 2084 |
| Israel | Hadera | 2084 |
| Israel | Haifa | 1536 |
| Israel | Jerusalem | 2084 |
| Israel | Kinneret | 2084 |
| Israel | Nazareth | 2084 |
| Israel | Petach Tiqwa | 2084 |
| Israel | Ramla | 2084 |
| Israel | Rehovot | 2084 |
| Israel | Tel Aviv | 2084 |
| Israel | Zefat | 2084 |
| Japan | Aichi | 2172 |
| Japan | Akita | 2172 |
| Japan | Aomori | 2172 |
| Japan | Chiba | 2172 |
| Japan | Ehime | 2172 |
| Japan | Fukui | 2172 |
| Japan | Fukuoka | 2172 |
| Japan | Fukushima | 2172 |
| Japan | Gifu | 2172 |
| Japan | Gunma | 2172 |
| Japan | Hiroshima | 2172 |
| Japan | Hokkaido | 2172 |
| Japan | Hyogo | 2172 |
| Japan | Ibaraki | 2172 |
| Japan | Ishikawa | 2172 |
| Japan | Iwate | 2172 |
| Japan | Kagawa | 2172 |
| Japan | Kagoshima | 2172 |
| Japan | Kanagawa | 2172 |
| Japan | Kochi | 2172 |
| Japan | Kumamoto | 2172 |
| Japan | Kyoto | 2172 |
| Japan | Mie | 2172 |
| Japan | Miyagi | 2172 |
| Japan | Miyazaki | 2172 |
| Japan | Nagano | 2172 |
| Japan | Nagasaki | 2163 |
| Japan | Nara | 2172 |
| Japan | Niigata | 2172 |
| Japan | Oita | 2172 |
| Japan | Okayama | 2172 |
| Japan | Okinawa | 2163 |
| Japan | Osaka | 2163 |
| Japan | Saga | 2163 |
| Japan | Saitama | 2172 |
| Japan | Shiga | 2172 |
| Japan | Shimane | 2163 |
| Japan | Shizuoka | 2172 |
| Japan | Tochigi | 2172 |
| Japan | Tokushima | 2172 |
| Japan | Tokyo | 2163 |
| Japan | Tottori | 2163 |
| Japan | Toyama | 2172 |
| Japan | Wakayama | 2172 |
| Japan | Yamagata | 2172 |
| Japan | Yamaguchi | 2163 |
| Japan | Yamanashi | 2172 |
| Norway | Akershus | 2154 |
| Norway | Aust-Agder | 2154 |
| Norway | Buskerud | 2154 |
| Norway | Finnmark | 2154 |
| Norway | Hedmark | 2154 |
| Norway | Hordaland | 2154 |
| Norway | Møre og Romsdal | 2148 |
| Norway | Nord-Trøndelag | 2148 |
| Norway | Nordland | 2154 |
| Norway | Oppland | 2154 |
| Norway | Oslo | 2148 |
| Norway | Rogaland | 2154 |
| Norway | Sør-Trøndelag | 2148 |
| Norway | Sognog Fjordane | 2154 |
| Norway | Telemark | 2154 |
| Norway | Trøndelag | 2148 |
| Norway | Troms | 2154 |
| Norway | Ukjent fylke | 2148 |
| Norway | Vest-Agder | 2154 |
| Norway | Vestfold | 2148 |
| Saudi Arabia | Al-Ahsaa | 1892 |
| Saudi Arabia | Al-Baha | 1892 |
| Saudi Arabia | Al-Joaf | 1892 |
| Saudi Arabia | Asir | 1892 |
| Saudi Arabia | Bisha | 1892 |
| Saudi Arabia | Eastern Province | 1892 |
| Saudi Arabia | Hafr Al-Batin | 1892 |
| Saudi Arabia | Hail | 1892 |
| Saudi Arabia | Jazan | 1892 |
| Saudi Arabia | Jeddah | 1892 |
| Saudi Arabia | Madinah | 1892 |
| Saudi Arabia | Makkah | 1892 |
| Saudi Arabia | Najran | 1892 |
| Saudi Arabia | Northern Borders | 1892 |
| Saudi Arabia | Qassim | 1892 |
| Saudi Arabia | Qunfotha | 1892 |
| Saudi Arabia | Qurayyat | 1892 |
| Saudi Arabia | Riyadh | 1892 |
| Saudi Arabia | Tabuk | 1892 |
| Saudi Arabia | Taif | 1892 |
| Sweden | Örebro | 2467 |
| Sweden | Östergötland | 2467 |
| Sweden | Blekinge | 2477 |
| Sweden | Dalarna | 2477 |
| Sweden | Gävleborg | 2467 |
| Sweden | Gotland | 2477 |
| Sweden | Halland | 2477 |
| Sweden | Jönköping | 2467 |
| Sweden | Jämtland | 2467 |
| Sweden | Kalmar | 2477 |
| Sweden | Kronoberg | 2477 |
| Sweden | Norrbotten | 2477 |
| Sweden | Södermanland | 2467 |
| Sweden | Skåne | 2467 |
| Sweden | Stockholm | 2477 |
| Sweden | Uppsala | 2477 |
| Sweden | Värmland | 2467 |
| Sweden | Västerbotten | 2467 |
| Sweden | Västernorrland | 2467 |
| Sweden | Västmanland | 2467 |
| Sweden | Västra Götaland | 2467 |
| United States | Alabama | 2628 |
| United States | Alaska | 2628 |
| United States | American Samoa | 2612 |
| United States | Arizona | 2628 |
| United States | Arkansas | 2628 |
| United States | California | 2628 |
| United States | Colorado | 2628 |
| United States | Commonwealth of Northern Mariana Islands | 2612 |
| United States | Connecticut | 2628 |
| United States | Delaware | 2628 |
| United States | District of Columbia | 2628 |
| United States | Florida | 2628 |
| United States | Georgia | 2628 |
| United States | Guam | 2612 |
| United States | Hawaii | 2628 |
| United States | Idaho | 2628 |
| United States | Illinois | 2628 |
| United States | Indiana | 2628 |
| United States | Iowa | 2628 |
| United States | Kansas | 2628 |
| United States | Kentucky | 2628 |
| United States | Louisiana | 2628 |
| United States | Maine | 2628 |
| United States | Maryland | 2628 |
| United States | Massachusetts | 2628 |
| United States | Michigan | 2628 |
| United States | Minnesota | 2628 |
| United States | Mississippi | 2628 |
| United States | Missouri | 2628 |
| United States | Montana | 2628 |
| United States | Nebraska | 2628 |
| United States | Nevada | 2628 |
| United States | New Hampshire | 2628 |
| United States | New Jersey | 2628 |
| United States | New Mexico | 2628 |
| United States | New York | 2628 |
| United States | New York City | 2612 |
| United States | North Carolina | 2628 |
| United States | North Dakota | 2628 |
| United States | Ohio | 2628 |
| United States | Oklahoma | 2628 |
| United States | Oregon | 2628 |
| United States | Pennsylvania | 2628 |
| United States | Puerto Rico | 2612 |
| United States | Rhode Island | 2628 |
| United States | South Carolina | 2628 |
| United States | South Dakota | 2628 |
| United States | Tennessee | 2628 |
| United States | Texas | 2628 |
| United States | U.S. Virgin Islands | 2612 |
| United States | Utah | 2628 |
| United States | Vermont | 2628 |
| United States | Virginia | 2628 |
| United States | Washington | 2628 |
| United States | West Virginia | 2628 |
| United States | Wisconsin | 2628 |
| United States | Wyoming | 2628 |

**Table S4**. Additional evaluation metrics for each model and disease combination forecasting the presence of disease for each region.

| **Disease** | **Model** | **F1-score** | **Precision** | **Recall** | **NPV** | **TN** | **FP** | **FN** | **TP** |
| --- | --- | --- | --- | --- | --- | --- | --- | --- | --- |
| **MERS** | XGB | 0.9 | 1.0 | 0.8 | 1.0 | 122 | 28 | 0 | 0 |
| **Q fever** | XGB | 0.8 | 0.7 | 0.9 | 0.9 | 94 | 41 | 7 | 84 |
| **Campylobacteriosis** | XGB | 1.0 | 0.9 | 1.0 | 0.9 | 17 | 19 | 2 | 236 |
| **Tularemia** | XGB | 0.7 | 0.6 | 0.9 | 1.0 | 124 | 32 | 6 | 52 |
| **Tick-borne Encephalitis** | XGB | 1.0 | 1.0 | 1.0 | 1.0 | 94 | 1 | 0 | 31 |
| **Brucellosis** | XGB | 0.7 | 0.5 | 0.9 | 0.9 | 137 | 73 | 11 | 87 |
| **MERS** | RF | 0.9 | 1.0 | 0.8 | 1.0 | 122 | 28 | 0 | 0 |
| **Q fever** | RF | 0.8 | 0.7 | 0.9 | 0.9 | 94 | 41 | 6 | 85 |
| **Campylobacteriosis** | RF | 1.0 | 0.9 | 1.0 | 0.9 | 17 | 19 | 1 | 237 |
| **Tularemia** | RF | 0.7 | 0.6 | 0.9 | 1.0 | 124 | 32 | 6 | 52 |
| **Tick-borne Encephalitis** | RF | 1.0 | 1.0 | 1.0 | 1.0 | 94 | 1 | 0 | 31 |
| **Brucellosis** | RF | 0.7 | 0.5 | 0.9 | 0.9 | 137 | 73 | 12 | 86 |
| **MERS** | LGBM | 0.9 | 1.0 | 0.8 | 1.0 | 122 | 28 | 0 | 0 |
| **Q fever** | LGBM | 0.8 | 0.7 | 0.9 | 0.9 | 94 | 41 | 6 | 85 |
| **Campylobacteriosis** | LGBM | 1.0 | 0.9 | 1.0 | 0.9 | 17 | 19 | 1 | 237 |
| **Tularemia** | LGBM | 0.7 | 0.6 | 0.9 | 1.0 | 124 | 32 | 6 | 52 |
| **Tick-borne Encephalitis** | LGBM | 1.0 | 1.0 | 1.0 | 1.0 | 94 | 1 | 0 | 31 |
| **Brucellosis** | LGBM | 0.7 | 0.5 | 0.9 | 0.9 | 137 | 73 | 11 | 87 |
| **MERS** | ARIMA | 1.0 | 1.0 | 1.0 | 1.0 | 143 | 7 | 0 | 0 |
| **Q fever** | ARIMA | 0.8 | 0.8 | 0.7 | 0.8 | 123 | 12 | 25 | 66 |
| **Campylobacteriosis** | ARIMA | 0.9 | 0.9 | 0.9 | 0.5 | 21 | 15 | 20 | 218 |
| **Tularemia** | ARIMA | 0.5 | 0.9 | 0.4 | 0.8 | 152 | 4 | 35 | 23 |
| **Tick-borne Encephalitis** | ARIMA | 0.7 | 1.0 | 0.5 | 0.9 | 95 | 0 | 15 | 16 |
| **Brucellosis** | ARIMA | 0.7 | 0.8 | 0.6 | 0.8 | 194 | 16 | 43 | 55 |
| **MERS** | SARIMAX | 1.0 | 1.0 | 0.9 | 1.0 | 137 | 13 | 0 | 0 |
| **Q fever** | SARIMAX | 0.7 | 0.7 | 0.9 | 0.9 | 94 | 41 | 13 | 78 |
| **Campylobacteriosis** | SARIMAX | 0.9 | 1.0 | 0.8 | 0.5 | 31 | 5 | 36 | 202 |
| **Tularemia** | SARIMAX | 0.7 | 0.6 | 0.9 | 0.9 | 124 | 32 | 7 | 51 |
| **Tick-borne Encephalitis** | SARIMAX | 1.0 | 1.0 | 0.9 | 1.0 | 94 | 1 | 2 | 29 |
| **Brucellosis** | SARIMAX | 0.7 | 0.5 | 0.9 | 0.9 | 136 | 74 | 14 | 84 |
| **MERS** | Prophet | 0.9 | 1.0 | 0.8 | 1.0 | 122 | 28 | 0 | 0 |
| **Q fever** | Prophet | 0.8 | 0.8 | 0.8 | 0.9 | 113 | 22 | 17 | 74 |
| **Campylobacteriosis** | Prophet | 1.0 | 1.0 | 1.0 | 0.8 | 30 | 6 | 9 | 229 |
| **Tularemia** | Prophet | 0.7 | 0.7 | 0.8 | 0.9 | 133 | 23 | 13 | 45 |
| **Tick-borne Encephalitis** | Prophet | 1.0 | 1.0 | 1.0 | 1.0 | 94 | 1 | 1 | 30 |
| **Brucellosis** | Prophet | 0.7 | 0.6 | 0.8 | 0.9 | 161 | 49 | 21 | 77 |
| **MERS** | RNN | 0.6 | 1.0 | 0.4 | 1.0 | 66 | 84 | 0 | 0 |
| **Q fever** | RNN | 0.7 | 0.5 | 0.9 | 0.9 | 60 | 75 | 8 | 83 |
| **Campylobacteriosis** | RNN | 0.9 | 0.9 | 1.0 | 0.8 | 14 | 22 | 3 | 235 |
| **Tularemia** | RNN | 0.5 | 0.4 | 0.8 | 0.9 | 70 | 86 | 10 | 48 |
| **Tick-borne Encephalitis** | RNN | 0.6 | 0.4 | 1.0 | 1.0 | 47 | 48 | 1 | 30 |
| **Brucellosis** | RNN | 0.6 | 0.4 | 0.9 | 0.9 | 78 | 132 | 9 | 89 |
| **MERS** | Transformer | 0.6 | 1.0 | 0.4 | 1.0 | 58 | 92 | 0 | 0 |
| **Q fever** | Transformer | 0.6 | 0.5 | 0.9 | 0.9 | 47 | 88 | 5 | 86 |
| **Campylobacteriosis** | Transformer | 0.9 | 0.9 | 1.0 | 0.9 | 6 | 30 | 1 | 237 |
| **Tularemia** | Transformer | 0.6 | 0.4 | 0.9 | 1.0 | 70 | 86 | 3 | 55 |
| **Tick-borne Encephalitis** | Transformer | 0.5 | 0.4 | 1.0 | 1.0 | 43 | 52 | 0 | 31 |
| **Brucellosis** | Transformer | 0.6 | 0.4 | 0.9 | 0.9 | 90 | 120 | 6 | 92 |
| **MERS** | LSTM | 0.6 | 1.0 | 0.4 | 1.0 | 65 | 85 | 0 | 0 |
| **Q fever** | LSTM | 0.6 | 0.5 | 0.8 | 0.8 | 63 | 72 | 14 | 77 |
| **Campylobacteriosis** | LSTM | 1.0 | 0.9 | 1.0 | 0.8 | 21 | 15 | 4 | 234 |
| **Tularemia** | LSTM | 0.5 | 0.4 | 0.8 | 0.9 | 73 | 83 | 12 | 46 |
| **Tick-borne Encephalitis** | LSTM | 0.6 | 0.4 | 0.9 | 1.0 | 56 | 39 | 2 | 29 |
| **Brucellosis** | LSTM | 0.6 | 0.4 | 0.8 | 0.9 | 92 | 118 | 16 | 82 |
